# Supplementary material for: Pyruvate Dehydrogenase Kinase 1 inhibition mediated oxidative phosphorylation enhancement in cartilage promotes osteoarthritis progression
Source: BMC Musculoskelet Disord. 2023 Jul 20;24:597. doi: 10.1186/s12891-023-06585-6 (PMC10357736; doi:10.1186/s12891-023-06585-6)
Supplement: Supplementary file 1 — Additional file 1. [file 12891_2023_6585_MOESM1_ESM.zip › MF.Gsea.1653627316242/GOMF_INOSITOL_PHOSPHATE_PHOSPHATASE_ACTIVITY.html]

Details for gene set GOMF\_INOSITOL\_PHOSPHATE\_PHOSPHATASE\_ACTIVITY[GSEA]

|  || Dataset | 55457\_collapsed\_to\_symbols.cls.cls #OA\_versus\_normal.cls.cls #OA\_versus\_normal\_repos |
| Phenotype | cls.cls#OA\_versus\_normal\_repos |
| Upregulated in class | normal |
| GeneSet | GOMF\_INOSITOL\_PHOSPHATE\_PHOSPHATASE\_ACTIVITY |
| Enrichment Score (ES) | -0.56166184 |
| Normalized Enrichment Score (NES) | -1.6514412 |
| Nominal p-value | 0.012027492 |
| FDR q-value | 0.13086244 |
| FWER p-Value | 0.967 |
Table: GSEA Results Summary

  

Fig 1: Enrichment plot: GOMF\_INOSITOL\_PHOSPHATE\_PHOSPHATASE\_ACTIVITY      
 Profile of the Running ES Score & Positions of GeneSet Members on the Rank Ordered List

  

| SYMBOL | TITLE | RANK IN GENE LIST | RANK METRIC SCORE | RUNNING ES | CORE ENRICHMENT || 1 | INPP5D | inositol polyphosphate-5-phosphatase D [Source:HGNC Symbol;Acc:HGNC:6079] | 3085 | 0.241 | -0.1869 | No |
| 2 | MTMR7 | myotubularin related protein 7 [Source:HGNC Symbol;Acc:HGNC:7454] | 4432 | 0.119 | -0.2655 | No |
| 3 | INPP4A | inositol polyphosphate-4-phosphatase type I A [Source:HGNC Symbol;Acc:HGNC:6074] | 5274 | 0.050 | -0.3199 | No |
| 4 | INPP5J | inositol polyphosphate-5-phosphatase J [Source:HGNC Symbol;Acc:HGNC:8956] | 6009 | -0.005 | -0.3758 | No |
| 5 | MINPP1 | multiple inositol-polyphosphate phosphatase 1 [Source:HGNC Symbol;Acc:HGNC:7102] | 6055 | -0.009 | -0.3774 | No |
| 6 | INPP4B | inositol polyphosphate-4-phosphatase type II B [Source:HGNC Symbol;Acc:HGNC:6075] | 6284 | -0.026 | -0.3894 | No |
| 7 | OCRL | OCRL inositol polyphosphate-5-phosphatase [Source:HGNC Symbol;Acc:HGNC:8108] | 7319 | -0.103 | -0.4472 | No |
| 8 | IMPA1 | inositol monophosphatase 1 [Source:HGNC Symbol;Acc:HGNC:6050] | 7332 | -0.105 | -0.4253 | No |
| 9 | INPP5F | inositol polyphosphate-5-phosphatase F [Source:HGNC Symbol;Acc:HGNC:17054] | 7734 | -0.136 | -0.4267 | No |
| 10 | INPP5K | inositol polyphosphate-5-phosphatase K [Source:HGNC Symbol;Acc:HGNC:33882] | 8103 | -0.163 | -0.4197 | No |
| 11 | INPP5A | inositol polyphosphate-5-phosphatase A [Source:HGNC Symbol;Acc:HGNC:6076] | 8813 | -0.218 | -0.4273 | No |
| 12 | INPP1 | inositol polyphosphate-1-phosphatase [Source:HGNC Symbol;Acc:HGNC:6071] | 10454 | -0.367 | -0.4746 | Yes |
| 13 | SYNJ1 | synaptojanin 1 [Source:HGNC Symbol;Acc:HGNC:11503] | 11577 | -0.512 | -0.4502 | Yes |
| 14 | IMPA2 | inositol monophosphatase 2 [Source:HGNC Symbol;Acc:HGNC:6051] | 11684 | -0.530 | -0.3431 | Yes |
| 15 | PTEN | phosphatase and tensin homolog [Source:HGNC Symbol;Acc:HGNC:9588] | 11836 | -0.556 | -0.2337 | Yes |
| 16 | INPP5E | inositol polyphosphate-5-phosphatase E [Source:HGNC Symbol;Acc:HGNC:21474] | 11882 | -0.568 | -0.1135 | Yes |
| 17 | INPP5B | inositol polyphosphate-5-phosphatase B [Source:HGNC Symbol;Acc:HGNC:6077] | 12723 | -0.884 | 0.0137 | Yes |
Table: GSEA details [plain text format]

  

Fig 2: GOMF\_INOSITOL\_PHOSPHATE\_PHOSPHATASE\_ACTIVITY      
 Blue-Pink O' Gram in the Space of the Analyzed GeneSet

  

Fig 3: GOMF\_INOSITOL\_PHOSPHATE\_PHOSPHATASE\_ACTIVITY: Random ES distribution      
 Gene set null distribution of ES for **GOMF\_INOSITOL\_PHOSPHATE\_PHOSPHATASE\_ACTIVITY**
